# Identification of glioblastoma-specific antigens expressed in patient-derived tumor cells as candidate targets for chimeric antigen receptor T cell therapy

**DOI:** 10.1093/noajnl/vdac177

**Published:** 2022-11-15

**Authors:** Tomoyoshi Nakagawa, Noriyuki Kijima, Kana Hasegawa, Shunya Ikeda, Moto Yaga, Tansri Wibowo, Tetsuro Tachi, Hideki Kuroda, Ryuichi Hirayama, Yoshiko Okita, Manabu Kinoshita, Naoki Kagawa, Yonehiro Kanemura, Naoki Hosen, Haruhiko Kishima

**Affiliations:** Department of Neurosurgery, Osaka University Graduate School of Medicine, Osaka, Japan; Department of Neurosurgery, Osaka University Graduate School of Medicine, Osaka, Japan; Laboratory of Cellular Immunotherapy, World Premier International Immunology Frontier Research Center, Osaka University, Osaka, Japan; Department of Functional Diagnostic Science, Osaka University Graduate School of Medicine, Osaka, Japan; Department of Hematology and Oncology, Osaka University Graduate School of Medicine, Osaka, Japan; Department of Hematology and Oncology, Osaka University Graduate School of Medicine, Osaka, Japan; Department of Neurosurgery, Osaka University Graduate School of Medicine, Osaka, Japan; Department of Neurosurgery, Osaka University Graduate School of Medicine, Osaka, Japan; Department of Neurosurgery, Osaka University Graduate School of Medicine, Osaka, Japan; Department of Neurosurgery, Osaka University Graduate School of Medicine, Osaka, Japan; Department of Neurosurgery, Osaka University Graduate School of Medicine, Osaka, Japan; Department of Neurosurgery, Osaka University Graduate School of Medicine, Osaka, Japan; Department of Biomedical Research and Innovation, Institute for Clinical Research, National Hospital Organization Osaka National Hospital, Osaka, Japan; Department of Neurosurgery, National Hospital Organization Osaka National Hospital, Osaka, Japan; Laboratory of Cellular Immunotherapy, World Premier International Immunology Frontier Research Center, Osaka University, Osaka, Japan; Department of Hematology and Oncology, Osaka University Graduate School of Medicine, Osaka, Japan; Department of Neurosurgery, Osaka University Graduate School of Medicine, Osaka, Japan

**Keywords:** B7-H3, CAR-T cell therapy, expression cloning, glioblastoma (GBM), monoclonal antibody

## Abstract

**Background:**

New therapies for glioblastoma (GBM) are urgently needed because the disease prognosis is poor. Chimeric antigen receptor (CAR)-T cell therapy that targets GBM-specific cell surface antigens is a promising therapeutic strategy. However, extensive transcriptome analyses have uncovered few GBM-specific target antigens.

**Methods:**

We established a library of monoclonal antibodies (mAbs) against a tumor cell line derived from a patient with GBM. We identified mAbs that reacted with tumor cell lines from patients with GBM but not with nonmalignant human brain cells. We then detected the antigens they recognized using expression cloning. CAR-T cells derived from a candidate mAb were generated and tested *in vitro* and *in vivo*.

**Results:**

We detected 507 mAbs that bound to tumor cell lines from patients with GBM. Among them, E61 and A13 reacted with tumor cell lines from most patients with GBM, but not with nonmalignant human brain cells. We found that B7-H3 was the antigen recognized but E61. CAR-T cells were established using the antigen-recognition domain of E61-secreted cytokines and exerted cytotoxicity in co-culture with tumor cells from patients with GBM.

**Conclusions:**

Cancer-specific targets for CAR-T cells were identified using a mAb library raised against primary GBM tumor cells from a patient. We identified a GBM-specific mAb and its antigen. More mAbs against various GBM samples and novel target antigens are expected to be identified using this strategy.

**Key Points**

- Monoclonal antibodies (E61, A13) that react to GBM cells were identified.
- GBM-specific antigen B7-H3 was recognized by E61.
- More targets are expected to be identified using this method.

**Importance of the Study**

The absence of highly specific target antigens for glioblastoma (GBM) cells is a barrier to the development of effective chimeric antigen receptor T cells to treat GBM. Our method of identifying novel GBM-specific targets should be useful to find those that are undetectable in comprehensive transcriptome analyses.

Glioblastoma (GBM) is one of the most frequent malignant brain tumors. Despite intensive treatment by surgery, radiation, and chemotherapy, the median survival is only approximately 15–20 months.^1,2^ Thus, more effective treatments are urgently needed, and various therapies for GBM are being tested or are in development^3–5^

One promising strategy against GBM is chimeric antigen receptor (CAR)-T cell therapy, which is effective against hematological malignancies.^6^ CAR-T cells targeting CD19 have generated unprecedented response rates in treating refractory B cell malignancies and became the first US Food and Drug Administration-approved cell-based therapy.^7–9^ Among solid cancers, GBM is a good candidate for CAR-T cell therapy. Indeed, early clinical trials of CAR-T cells directed to interleukin-13 receptor alpha 2 (IL13Rα2), epidermal growth factor receptor variant III (EGFRvIII), and human epidermal growth factor receptor 2 (HER2), have generated promising results against brain tumors including GBM.^10–14^ In addition, CAR-T cells targeting B7-H3 have been developed for GBM,^4,5,15^ pediatric brain tumors,^16^ and skull base chordoma.^17^ However, CAR-T cell therapy is more effective against hematological cancers than against GBM. One of the major reasons for this is that only a few target antigens are highly specific for GBM cells. The highly specific, aberrantly spliced variant EGFRvIII is expressed only in a subset of GBM tumor cells.^13,18^ In addition, immunotherapy targeting a single antigen frequently causes immune evasion by antigen loss in tumor cells.^11,19^ Therefore, more target antigens specifically expressed in GBM tumor cells are needed. Most transcripts or proteins highly specific for GBM cells have been identified by extensive transcriptome or proteome analyses.^20,21^ Consequently, identifying novel cell surface target antigens is complicated. However, cancer-associated antigen epitopes formed by posttranslational protein modifications such as glycosylation,^22–24^ or conformational changes,^25^ might have been missed. Therefore, novel antigens targeting cell surfaces could still be discovered by thoroughly screening cancer-specific mAbs and characterizing their target antigens. In fact, we applied this strategy to multiple myeloma (MM)-specific antigens and recently identified a mAb that reacts with MM-specific glycoforms of CD98 heavy chain.^26^

Here, we established mAbs that reacted with tumor cells derived from a patient (PDTCs) with GBM using a serum-free culture medium containing EGF and basic Fibroblast Growth Factor (bFGF). Although this medium was originally developed to culture neural stem cells,^27,28^ the characteristics of primary GBM were retained to some extent.^29–31^ We then searched for mAbs that would react with PDTCs from patients with GBM but not from nonmalignant brain cells and identified the antigens they recognized. Finally, we generated T cells transduced with CAR derived from a candidate mAb and tested its efficacy in vitro and in vivo.

## Material and Methods

### Human Tissues and Cells

This study complied with the principles of the Helsinki Declaration (2013 amendment) and was approved by the Ethics Review Board at Osaka University (Approval no: 20561). We obtained written informed consent from patients with GBM to obtain surgically excised brain tumor tissues at Osaka University Hospital (GDC519, GDC1520, GDC3320, GDC521) and Osaka National Hospital (GDC40), and from those with refractory epilepsy treated by anterior temporal lobectomy to collect non-tumorous brain tissues.

### Animal Experiments

Six-week-old BALB/c mice (CLEA Japan) were purchased from CLEA Japan Inc., and NOD/Shi-scid IL2Rγ KO mice (NOG) were purchased from Central Institute for Experimental Animals (CIEA). All animal experiments were performed with the approval of the Institutional Animal Care and Use Committee at the Osaka University Medical School (approval numbers: 03-071-000 and 04-028-002). All procedures involving animals were performed according to the animal use guidelines of the Animal Experiment Committee of Osaka University.

### Establishment of GBM-PDTCs From Surgical Specimens and Preparation of Non-tumorous Brain Tissues

We obtained GDC40^32,33^ which is an established GBM PDTC and GDC519 from freshly dissected samples from patients with GBM. GBM tumor tissues were mechanically minced using scalpels and scissors, then digested by shaking in 0.05% trypsin-EDTA (Thermo Fisher Scientific Inc.) at 37°C for 30–60 min. Trypsin inhibitor (Roche Diagnostics), DNase I (Thermo Fisher Scientific Inc.) and base medium (DMEM/F12 containing 15 mM HEPES [Wako Pure Chemical Industries]) and 1% Antibiotic-Antimycotic (Thermo Fisher Scientific Inc.) were added to the tissues and centrifuged at 180 × *g* for 5 min. Pelleted cells were incubated in cell culture flasks with serum-free culture medium at 37°C under a 5% CO_2_ atmosphere. Serum-free DMEM/Ham’s F12 culture medium (Wako Pure Chemical Industries) contained 20 ng/ml EGF (PeproTech), 20 ng/ml bFGF (PeproTech), 5 µg/ml heparin (Sigma-Aldrich Corp.), 10 ng/ml leukemia inhibitory factor (Merck Millipore), and 2% B27 supplements (Thermo Fisher Scientific Inc.). Human non-tumorous brain tissues were similarly processed as described above.

### Immunization of Mice and Generation of Hybridomas With GBM-PDTC

Six-week-old BALB/c mice (CLEA Japan) were immunized by injecting their footpads with 1 × 10^6^ GBM-PDTCs (GDC40) weekly for 4 weeks then euthanizing them by CO_2_ inhalation. Popliteal lymph nodes were removed, and lymphocytes were extracted and fused with SP2/0 mouse myeloma cells using polyethylene glycol (Roche Applied Science, Penzberg, Germany). The hybridoma cells were suspended and incubated in 96 well plates. We dissociated GDC40 using TrypLE Select (Thermo Fisher Scientific Inc.), blocked nonspecific binding with Human Serum AB (Gemini Bio-Products), then incubated hybridoma supernatants with phycoerythrin (PE)-conjugated anti-mouse Immunoglobulin G (IgG) antibody (eBioscience), and analyzed the cells by flow cytometry. Hybridoma clones producing mAbs that reacted with GDC40 were selected, stained at room temperature, and analyzed using a FACSCanto II flow cytometer (BD Biosciences). Data were analyzed using the FlowJo software.

### Flow Cytometry

Single-cell suspensions of PTDCs or non-tumorous brain tissues were stained with fluorochrome-conjugated mAbs, and PDTCs were dissociated with TrypLE Select. Single-cell suspensions were prepared from the tissues of 2 patients with refractory epilepsy that was treated by anterior temporal lobectomy. Tissue samples were immediately dissociated by enzymic digestion using Brain Tumor Dissociation Kits (P) and GentleMACS Octo Dissociator with Heaters (Miltenyi Biotec) as described by the manufacturer. The resultant cell suspensions were passed through a 70-µm filter with HBSS with Ca^2+^ and Mg^2+^ (Sigma Aldrich Corp.) and centrifuged for 5 min at 180 × *g*. The filtered cells were lysed with ACK buffer (Thermo Fisher Scientific Inc.) to remove red blood cells and resuspended in a staining medium (2% Fetal Bovine Serum (FBS) + Phosphate Buffered Saline (PBS).

Resuspended cells were incubated with Human Serum AB to block nonspecific binding followed by the established mAbs. The cells were stained at room temperature in the following order: PE-conjugated anti-mouse IgG antibody, fluorochrome-conjugated anti-CD31-APC (eBioscience), anti-CD45-FITC (BioLegend), and propidium iodide, then analyzed by flow cytometry using the FACSCanto Ⅱ. Data were analyzed using FlowJo software.

### Expression Cloning

Expression cloning proceeded as described.^34^ A cDNA library was generated from GDC40 using Superscript Choice System (Invitrogen) and linked with a BstXI adaptor. Complementary DNA fragments (1.0–5.0 kb) were selected using CHROMA SPIN columns (Takara Bio Inc.), purified by agarose gel electrophoresis, then subcloned into retroviral vector pMXs (a kind gift from Toshio Kitamura, University of Tokyo). The cDNA library constructed from the GDC40 cells was transduced into BaF3 cells, which were incubated with candidate mAbs and analyzed by fluorescence-activated cell sorting (FACS). BaF3 cells that reacted against E61 were sorted and expanded, then DNA extracted using DNeasy Blood and Tissue kits (Qiagen, GmbH) was amplified by PCR and then analyzed by Sanger sequencing.

### Development of Chimeric-antigen-receptor T Cells for the Candidate Antigen

Antibody variable regions (VH and VL) of the hybridoma obtained by 5′-RACE PCR using Smarter RACE PCR kits (Takara Bio) were sequenced. Isolated cDNAs of the variable regions of the heavy and κ light chains were fused with CD28 and CD3ζ cDNAs by overlapping PCR as described.^35^ The resultant CAR construct was inserted into the pMXs retroviral vector. We generated a viral supernatant by transfecting 293T cells co-transfected with the retroviral vector, gag-pol, and VSV-G envelope plasmids with Lipofectamine 2000 (Thermo Fisher Scientific Inc.). Supernatants containing the retrovirus were collected 48 h and 72 h later, then transduced with the retroviral vector. Peripheral blood mononuclear cells (PBMCs) activated with anti-CD3 (OKT3, eBioscience) and anti-CD28 (CD28.2, eBioscience) were incubated for 1 day, then recombinant human interleukin (IL)-2 (Shionogi Pharma) was added to the culture at a final concentration of 100 IU/ml. Two days later, PBMCs were transduced with the CAR retrovirus using RetroNectin (Takara Bio Inc.). The transduced cells were cultured with IL-2 (100 IU/ml) for 8–10 days. The cells were stained with goat anti-mouse F(ab′)2-Alexa Fluor 647 (Jackson ImmunoResearch Laboratories Inc.), then the transduction efficiency of CAR was determined using the FACSCanto II.

### Cytokine Release Assays

Cytokine expression by CAR-T or control T cells co-cultured with target cells was assessed using Quantikine ELISA kits (IL-2 and interferon gamma [IFN-γ]; R&D Systems Inc.). Effector and target cells (1 × 10^5^ each at an effector/target [E/T]) ratio of 1 were co-cultured for 24 h in technical-triplicate wells. The secretion of IL-2 and IFN-γ was measured in culture supernatants diluted to fall within the linear assay range.

### ^51^Cr Cytotoxicity Assay

The ability of the CAR-T cells to lyse tumor cells was assessed as ^51^Cr release. Briefly, 1 × 10^6^ target cells were labeled for 2 h at 37°C with 200 μCi of [^51^Cr] sodium chromate (GE Healthcare). Labeled target cells (1 × 10^4^) were incubated with effector cells for 4 h at E/T ratios of 1 and 10. The amount of ^51^Cr released in harvested supernatants was determined using a gamma counter. Maximum and spontaneous ^51^Cr release was determined by incubating 1 × 10^4^ labeled targets in either 1% Triton X-100 or culture medium in technical triplicate wells. Percentage-specific lysis was calculated as: [(specific ^51^Cr release − spontaneous ^51^Cr release)/(maximum ^51^Cr release − spontaneous ^51^Cr release)] × 100.

### Patient-Derived Orthotopic Xenograft Model

We established patient-derived orthotopic xenografts using NOD/Shi-scid IL2Rγ KO mice (NOG) (CIEA, Kawasaki, Japan). The mice were anesthetized with isoflurane, then 2 × 10^5^ GDC40 and GDC519 cells labeled with GFP and luciferase in 2 μl of PBS were introduced into the right cerebrum using a stereotactic injector (Muromachi) 1 mm forward of the bregma, 2 mm right, 2 mm deep. Two weeks thereafter, we injected CAR-T cells into the same point. We monitored tumor progression or regression using an In Vivo Imaging System(PerkinElmer Inc.) at 1, 2, 3, 5, 8, and 10 weeks after the tumor injections. The mice were sacrificed when neurological symptoms developed. Mouse survival was analyzed using Kaplan–Meier curves and differences were assessed using log-rank tests.

### Statistical Analysis

Differences between samples were determined using unpaired two-tailed Student *t-*tests with *P* < .05 indicating a significant difference. Data were statistically analyzed using JMP Pro 13 (JMP Software).

## Results

### Establishment of GBM-PDTC

**Figure 1.**
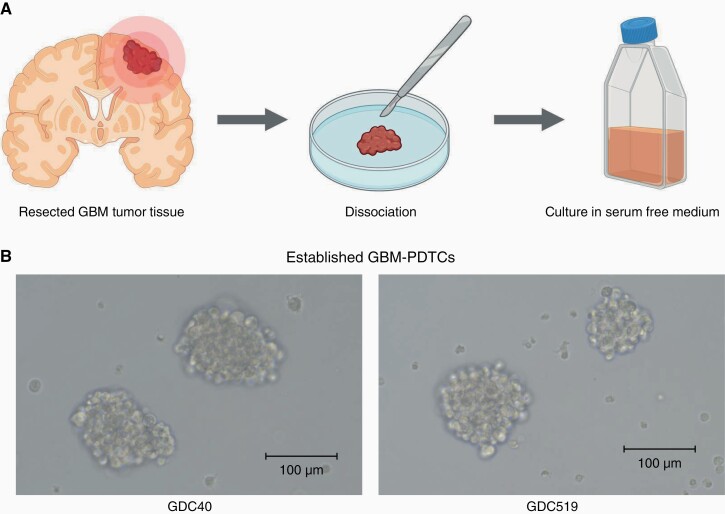
Establishment of PDTCs from patients with GBM. (A) Establishment of PDTCs from resected GBM specimens. (B) Representative morphology of PDTCs GDC40 and GDC519. GBM, glioblastoma; PDTCs, tumor cells derived from a patient.

The GBM-PDTC lines, GDC40^32,33^ and GDC519, were established from 2 patients with GBM. Briefly, GDC40 was established from a 69-year-old female patient with isocitrate dehydrogenase 1 (IDH1) wild type, *O*^6^-methylguanine-methyltransferase promoter methylation (MGMT) methylated, and telomerase reverse transcriptase (TERT) promotor mutant GBM using the neurosphere technique with the serum-free culture medium developed to isolate normal human neural stem/progenitor cells.^36^ The GDC519 cell line was derived from a 66-year-old male patient with the IDH1 wild type, MGMT unmethylated, and TERT promotor mutant GBM (Figure 1A). Both GBM-PDTC lines were cultured as floating tumor spheres in flasks (Figure 1B).

### Generation of mAb Library Against GBM-PDTCs

We immunized BALB/c mice with GDC40 cells and generated hybridomas by fusing the lymphocytes with SP2/0 mouse myeloma cells. We then selected hybridomas producing mAbs that bound to GDC40 cells by staining them with hybridoma supernatant and generated 507 clones of mAbs that recognized GDC40 cells.

### Identification of GBM-specific mAbs, E61, and A13

**Figure 2.**
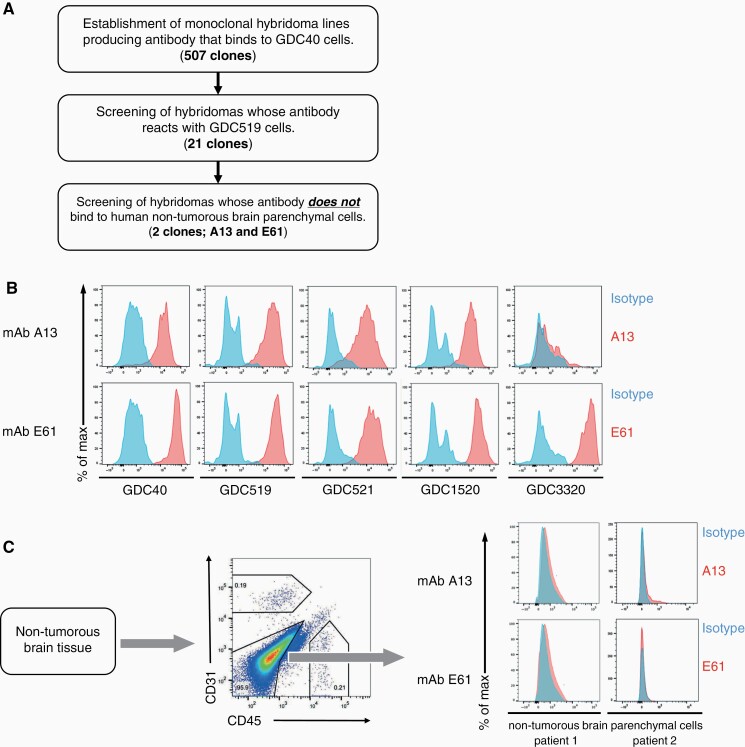
Identification of A13 and E61 as GBM-specific mAbs. (A) Screening procedure. (B) Flow cytometry of A13 or E61 mAb binding to PDTCs derived from 5 patients. Blue histogram, isotype control. (C) Representative flow cytometry findings of A13 or E61 mAb binding to nonmalignant brain parenchymal cells from patients with epilepsy treated by temporal lobectomy (*n* = 2). Blue histogram, isotype control. GBM, glioblastoma; PDTCs, tumor cells derived from a patient.

Among the 507 mAbs that reacted with GDC40 cells, we selected 21 that were also bound to GDC519 cells. We excluded the possibility of mAbs reacting with nonmalignant brain tissues as follows. Single-cell suspensions were prepared from the brain tissues of 2 patients with refractory epilepsy treated by anterior temporal lobectomy. The cells were stained with the candidate mAbs and analyzed by flow cytometry. The candidates A13 and E61 bound to both GBM PDTCs, but not to CD45^-^CD31^-^ cells, most of which were neurons, astrocytes, and oligodendrocytes^37^ in nonmalignant, lateral temporal lobe tissues from patients with epilepsy (Figure 2A–C). E61 was also bound to the other 3 GBM PDTCs examined, whereas A13 reacted with 2 (Figure 2B).

### E61 Recognized B7-H3

**Figure 3.**
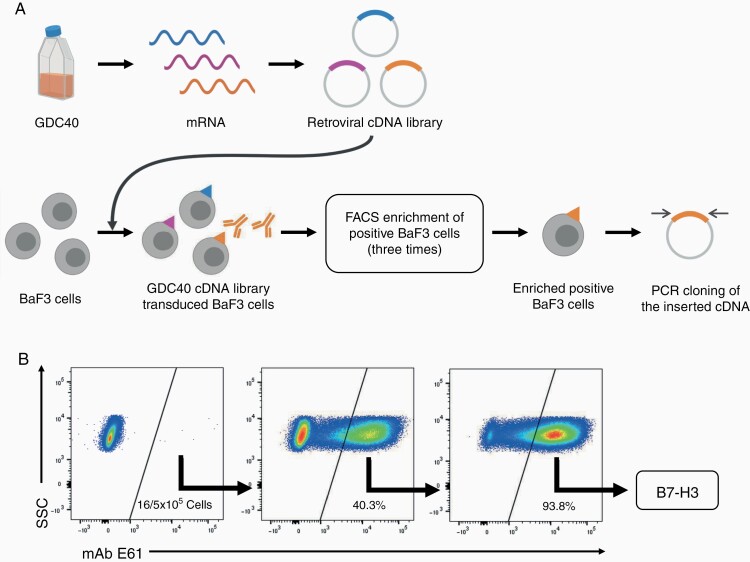
E61 recognizes B7-H3. (A) Expression cloning to identify antigens recognized by mAbs. (B) Flow cytometry of E61-positive cell enrichment in expression cloning of E61 antigen.

We identified the antigen recognized by E61 using expression cloning (Figure 3A). A cDNA library of GDC40 cells was established and cloned into the pMXs retroviral vector. The retroviral cDNA library was transduced into mouse BaF3 cells, which did not react with E61. Transfected BaF3 cells were stained with E61, and cells expressing the antigen recognized by E61 were enriched by FACS. After 3 rounds of sorting, all cells were positively stained with E61 (Figure 3B). Finally, PCR amplification and cDNA sequencing of the E61-positive BaF3 cells revealed that E61 reacted with B7-H3. We also attempted to identify the antigen recognized by A13 using expression cloning. However, BaF3 cells reacting with A13 could not be enriched by FACS, suggesting that the epitope recognized by A13 was not formed in mouse BaF3 cells expressing human cDNA derived from GDC40 cells.

### E61-Derived CAR-T Cells Recognized and Killed GBM-PDTCs In Vitro

**Figure 4.**
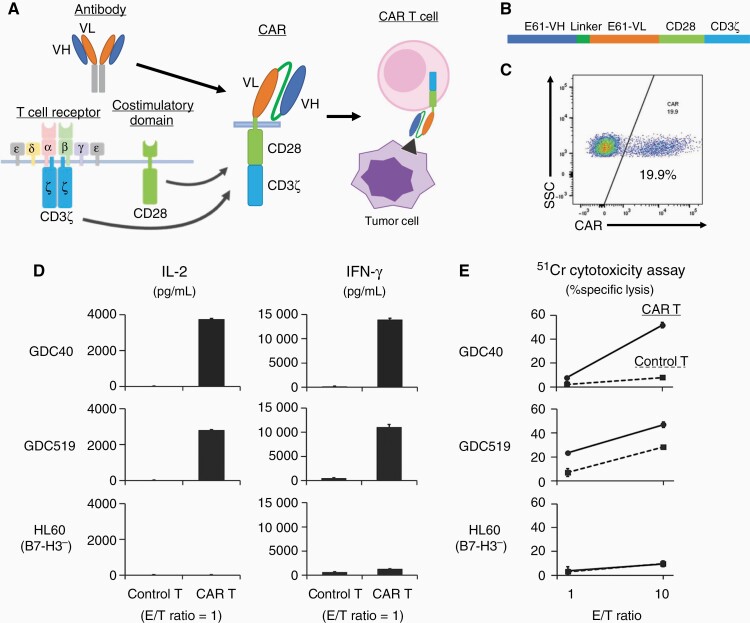
E61 mAb derived from CAR-T cells specifically recognizes and kills GBM cells expressing B7-H3. (A) Establishment of CAR-T cells using E61 mAb. (B) Construct of E61 CAR. (C) Transduction efficiency of E61 CAR-T cells into human T cells. (D) Interleukin (IL)-2 and Interferon gamma (IFN-γ) secretion by CAR-T cells measured 24 h after co-culture with tumor cells (E/T ratio = 1). (E) Assay of ^51^Cr release to measure specific lysis of target cells by CAR-T cells. Controls were mock-transduced T cells. GBM, glioblastoma; CAR-T cells, chimeric antigen receptor T cells.

The variable regions of E61 were sequenced using 5′ RACE PCR. A DNA construct expressing the E61 mAb derived from CAR was generated by fusing the variable region of E61 with the cytoplasmic region of CD28 and CD3ζ. Thereafter, E61-CAR-T cells were established by transducing the CAR construct into T cells activated by CD3 and CD28 mAbs (Figure 4A–C). The E61-CAR-T cells produced IFN-γ and IL-2 when co-cultured with GDC40 or GDC519 whereas non-transduced T cells did not (Figure 4D). In addition, E61-CAR-T cells were significantly cytotoxic against GDC40 and GDC519 (Figure 4E). In contrast, co-culture with HL-60 cells that do not express B7-H3, did not result in cytokine expression or cytotoxicity in E61-CAR-T cells. These results suggest that E61-CAR-T cells specifically recognize cells expressing B7-H3 and are cytotoxic. However, we could not show the anti-GBM effect in vivo (Supplementary Figure 1).

The variable regions of A13 were also sequenced and CAR derived from A13 was similarly generated (Supplementary Figure 2A and B). A13-CAR-T cells co-cultured with both GBM PDTCs did not produce IL-2 or IFN-γ and were not significantly cytotoxic (Supplementary Figure 2C and D).

## Discussion

Here, we established 507 mAbs that reacted with GBM cells. Instead of using GBM tumor cell lines such as U87 to immunize the mice,^38,39^ we used PDTCs from a patient with GBM. The GBM PDTCs retained some of the biological features of the GBM tumor cells derived from patients.^29,31,40–42^ We used PDTCs from one patient as an immunogen and from 5 others to examine the reactivity of the candidate mAbs. However, various antigens that are expressed in primary GBM cells might be isolated using PDTCs from various patients as immunogens and examining more patients with GBM. In addition, GBM PDTCs established in serum-free cell culture medium containing EGF and bFGF have the features of GBM stem-like cells.^27,28,41,42^ Thus, our strategy might be used to isolate mAbs that react with GBM stem-like cells.

One of the 2 GBM -specific mAbs, E61, recognized B7-H3. We also showed that E61 derived from CAR-T cells was cytotoxic against GBM-PDTCs in vitro. B7-H3 is one of the most promising target antigens for CAR-T cells against GBM.^4,5^ In addition, B7-H3 is abundantly expressed and is thus a good target for CAR-T cell therapy in brain tumors other than GBM^43,44^ and various solid tumors.^16,45^ Hayder *et al.* recently found that the most frequently expressed antigen in pediatric brain tumors (35 ependymomas and 14 high-grade gliomas) was B7-H3 compared with GD2, IL-13Rα2, EphA2, and HER2.^46^ Clinical trials of CAR-T cell therapy targeting B7-H3 are underway.^15^ Our results support the notion that B7-H3 is an excellent target for CAR-T cell therapy against GBM. However, the absence of E61-CAR- T cell effects in vivo suggests that previous CAR constructs used to target B7-H3 might be more effective than E61-CAR.^4,5^ Because B7-H3 expressed in pericytes and myeloid cells^47^ might cause on-target off-tumor side effects, care is needed in clinical trials of CAR-T cells targeting B7-H3. The splice variant 4Ig-B7H3 is specific to GBM cells, suggesting that 4Ig-B7H3 is a more specific target for CAR-T cells.^47^

We could not identify the antigen for A13 by expression cloning, because A13 did not react with any BaF3 cells expressing the cDNA library of GBM cells. Expression cloning works only when epitopes recognized by a mAb are formed in mouse cells expressing human cDNA. A13 might recognize an epitope that is not formed in mouse cells, for example, glycan-associated antigens. Another possibility is that A13 recognizes human leukocyte antigen (HLA) molecules because GBM and nonmalignant brain cells were derived from different individuals. HLA is expressed as a heterodimer of an α chain and β2 micro-globulin in class I HLA or as α and β chains in class II HLA. Neither an α, nor a β chain can be solely expressed on the surface of mouse BaF3 cells, and therefore cannot be identified as an antigen by expression cloning. Antigens recognized by A13 should be identified by other methods, for example, mass spectrometry of immune precipitates with A13.

Our study has some limitations. Unlike hematological cancers, de-selection with nonmalignant brain cells is difficult. We did not generate many GBM-specific antibodies. Other strategies such as phage-display technology^48^ could be applied to identify GBM-specific mAbs, although phage-display using whole cell panning is complicated by low target antigen density, high background due to irrelevant antigens, and nonspecific binding of phage particles to cell surfaces.^49^ We obtained antibodies of mouse origin that might not be optimal for the generation of CAR-T cells for infusion in humans.

In summary, our method of identifying cell surface target antigens using patient-derived tumor specimens^25,26^ is useful not only for hematological cancers but also for solid tumors such as GBM. The fact that we could isolate B7H3 as a candidate antigen is proof of concept. Although we have not yet identified a novel target antigen, we are presently generating more mAbs and screening those that are GBM-specific.

## Supplementary Material

vdac177_suppl_Supplementary_Figure_S1

vdac177_suppl_Supplementary_Figure_S2

vdac177_suppl_Supplementary_Data
