# Supplementary material for: Identification of glioblastoma-specific antigens expressed in patient-derived tumor cells as candidate targets for chimeric antigen receptor T cell therapy
Source: Neurooncol Adv. 2022 Nov 15;5(1):vdac177. doi: 10.1093/noajnl/vdac177 (PMC9798403; doi:10.1093/noajnl/vdac177)
Supplement: vdac177_suppl_Supplementary_Figure_S1 [file vdac177_suppl_supplementary_figure_s1.pptx]

## Slide 1
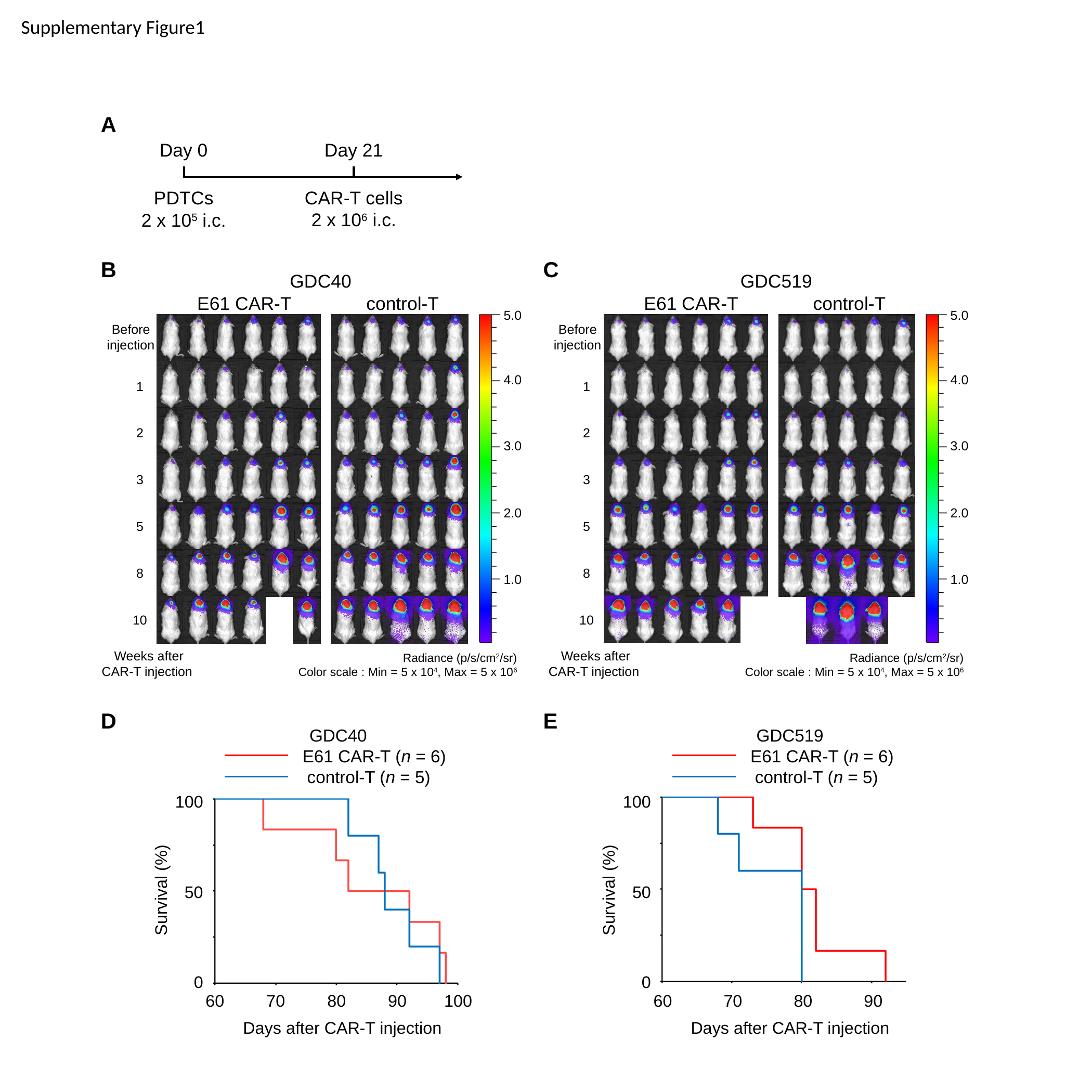

Supplementary Figure1
A
Day 0
Day 21
CAR-T cells
2 x 10^6^ i.c.
PDTCs
2 x 10^5^ i.c.
B
C
 GDC40
E61 CAR-T　 　control-T
1
2
3
5
8
10
Before
injection
 Weeks after
CAR-T injection
Radiance (p/s/cm2/sr)
Color scale : Min = 5 x 10^4^, Max = 5 x 10^6^
5.0
4.0
3.0
2.0
1.0
　GDC519
E61 CAR-T　 　control-T
5.0
4.0
3.0
2.0
1.0
1
2
3
5
8
10
Before
injection
 Weeks after
CAR-T injection
Radiance (p/s/cm2/sr)
Color scale : Min = 5 x 10^4^, Max = 5 x 10^6^
E
D
 　　　 GDC40
　　　　　　E61 CAR-T (n = 6)
　　　　　　 control-T (n = 5)
100
50
0
Survival (%)
60 70 80 90 100
 Days after CAR-T injection
 　　　　 GDC519
　　　　　　E61 CAR-T (n = 6)
　　　　　　 control-T (n = 5)
100
50
0
Survival (%)
60 70 80 90
 Days after CAR-T injection
