# Supplementary material for: Identification of glioblastoma-specific antigens expressed in patient-derived tumor cells as candidate targets for chimeric antigen receptor T cell therapy
Source: Neurooncol Adv. 2022 Nov 15;5(1):vdac177. doi: 10.1093/noajnl/vdac177 (PMC9798403; doi:10.1093/noajnl/vdac177)
Supplement: vdac177_suppl_Supplementary_Figure_S2 [file vdac177_suppl_supplementary_figure_s2.pptx]

## Slide 1
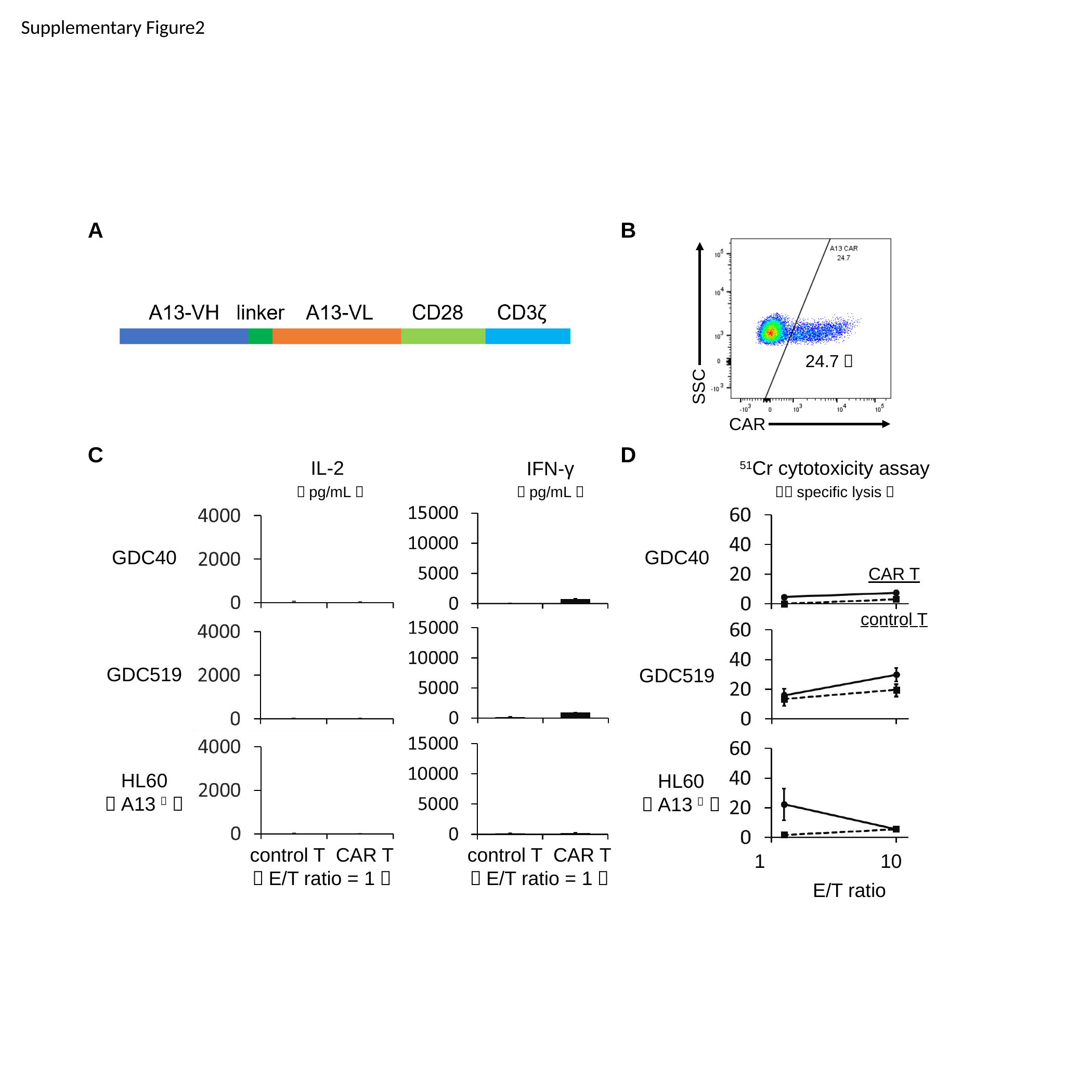

Supplementary Figure2
A
B
SSC
CAR
24.7％
C
D
51Cr cytotoxicity assay
（％specific lysis）
CAR T
control T
IL-2
（pg/mL）
IFN-γ
（pg/mL）
GDC40
GDC519
HL60
（A13ー ）
control T CAR T
（E/T ratio = 1）
control T CAR T
（E/T ratio = 1）
GDC40
GDC519
HL60
（A13ー ）
10
1
E/T ratio
