# Supplementary material for: Identification of glioblastoma-specific antigens expressed in patient-derived tumor cells as candidate targets for chimeric antigen receptor T cell therapy
Source: Neurooncol Adv. 2022 Nov 15;5(1):vdac177. doi: 10.1093/noajnl/vdac177 (PMC9798403; doi:10.1093/noajnl/vdac177)
Supplement: vdac177_suppl_Supplementary_Data [file vdac177_suppl_supplementary_data.docx]

**Supplementary Figure 1. CAR-T cells derived from A13 mAb do not express cytokines or exert cytotoxicity against GBM cells.**

(A) Construction of A13 CAR. (B) Transduction efficiency of A13 CAR-T into human T cells. (C) Interleukin (IL)-2 and Interferon gamma (IFN-γ) secretion by A13 CAR-T cells measured after 24 h of co-culture with tumor cells (E/T ratio = 1). (D) Assay of ^51^Cr release to determine specific lysis of target cells by CAR-T cells. Controls were mock-transduced T cells.

**Supplementary Figure 2. B7-H3 CAR-T cells did not exert anti-GBM effects in vivo.**

(A) Experimental design of B-E (B and C) Bioluminescence imaging of mice at 1, 2, 3, 5, 8, 10 weeks after injection with B7-H3 CAR-T and control CAR-T cells. Mice transplanted with (B) GDC40 and (C) GDC519. (D and E) Survival curves of mice injected with B7-H3 CAR-T and control CAR-T cells.
